# Systematic characterization of autophagy-related genes during the adipocyte differentiation using public-access data

**DOI:** 10.18632/oncotarget.24506

**Published:** 2018-02-15

**Authors:** Mahmoud Ahmed, Huynh Quoc Nguyen, Jin Seok Hwang, Sahib Zada, Trang Huyen Lai, Sang Soo Kang, Deok Ryong Kim

**Affiliations:** ^1^ Department of Biochemistry and Convergence Medical Sciences and Institute of Health Sciences, Gyeongsang National University School of Medicine, Jinju 527-27, Republic of Korea; ^2^ Department of Anatomy and Convergence Medical Sciences and Institute of Health Sciences, Gyeongsang National University School of Medicine, Jinju 527-27, Republic of Korea

**Keywords:** autophagy, adipocyte differentiation, 3T3-L1, RNA-seq, gene ontology

## Abstract

Autophagy contributes to reorganizing intracellular components and forming fat droplets during the adipocyte differentiation. Here, we systematically describe the role of autophagy-related genes and gene sets during the differentiation of adipocytes. We used a public dataset from the European Nucleotide Archive from an RNA-seq experiment in which 3T3-L1 cells were induced by a differentiation induction medium, total RNA was extracted and sequenced at four different time points. Raw reads were aligned to the UCSC mouse reference genome (mm10) using HISAT2, and aligned reads were summarized at the gene or exon level using HTSeq. DESeq2 and DEXSeq were used to model the gene and exon counts and test for differential expression and relative exon usage, respectively. After applying the appropriate transformation, gene counts were used to perform the gene set and pathway enrichment analysis. Data were obtained, processed and annotated using R and Bioconductor. Several autophagy-related genes and autophagy gene sets, as defined in the Gene Ontology, were actively regulated during the course of the adipocyte differentiation. We further characterized these gene sets by clustering their members to a few distinct temporal profiles. Other potential functionally related genes were identified using a machine learning procedure. In summary, we characterized the autophagy gene sets and their members to biologically meaningful groups and elected a number of genes to be functionally related based on their expression patterns, suggesting that autophagy plays a critical role in removal of some intracellular components and supply of energy sources for lipid biogenesis during adipogenesis.

## INTRODUCTION

3T3-L1 pre-adipocyte is a mouse fibroblast that has the potential to differentiate into a mature adipocyte when treated with the MDI (160 nM insulin, 250 nM dexamethasone, and 0.5 mM 1-methyl-3-isobutylxanthine) differentiation induction medium [1]. Therefore, MDI-induced 3T3-L1 cells can be used as a platform for studying the adipocyte differentiation and further understanding the development of obesity and insulin resistance. Autophagy, a lysosome-dependent degradation process, is involved in many cellular mechanisms including stress response, cell growth and death, and cell differentiation. Particularly, it contributes to reorganizing intracellular components and forming fat droplets during the adipocyte differentiation [2]. Indeed, inhibition of autophagy during the adipogenic induction of 3T3-L1 cells results in the prevention of formation of lipid droplets [3].

High through-put technologies such as microarrays and RNA-seq facilitate the study of the cell biology by enabling the investigation of a large number of genetic elements simultaneously compared to the conventional laboratory techniques. The availability of public-access data in repositories such as the Sequence Read Archive (SRA) and the European Nucleotide Archive (ENA) enables biologists to reexamine previously published data for their own purposes of discovery and hypothesis generation [4, 5].

**Figure 1:**
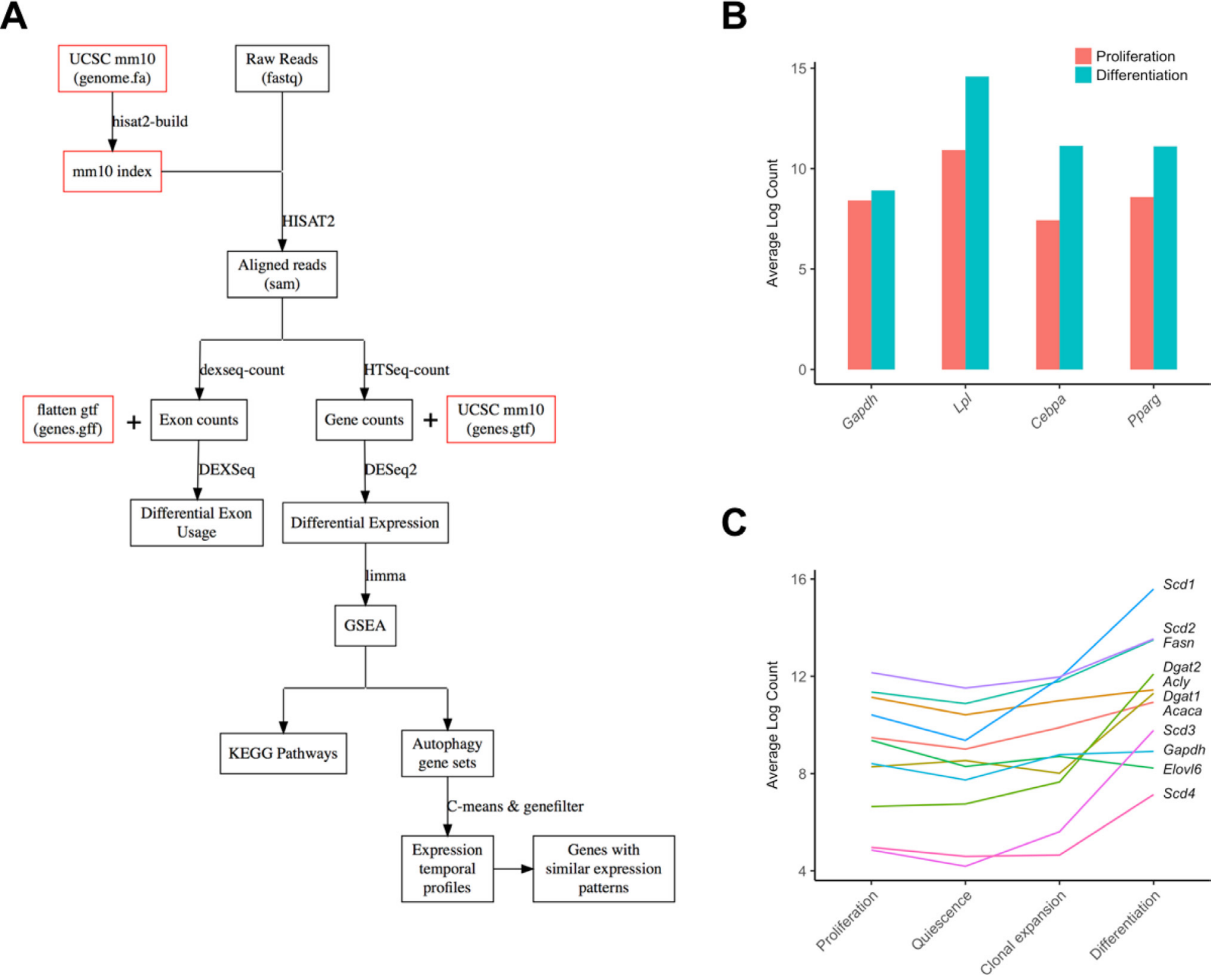
Analysis workflow and characterization of adipogenic and lipogenic markers (**A**) A digram of the overall workflow of the study. (**B**) Characterization of three adipocyte differentiation markers and a control in two developmental stages (control proliferating cells and differentiating cells) are shown as bars. (**C**) Characterization of ten lipogenesis markers and a control in four developmental stages (proliferation, quiescence, clonal expansion, and differentiation) were shown as individual lines. *Gapdh*; Glyceraldehyde-3-Phosphate Dehydrogenase, *Cebpa*, CCAAT/enhancer binding protein (C/EBP), alpha; *Lpl*, lipoprotein lipase; *Pparg*, peroxisome proliferator activated receptor gamma, *Acaca*, Acetyl-CoA Carboxylase Alpha; *Acly*, ATP Citrate Lyase; *Dgat*, Diacylglycerol O-Acyltransferase; *Elov6*, Fatty Acid Elongase 6; *Fasn*, Fatty Acid Synthase; *Scd*, Stearoyl-CoA Desaturase.

Here, we use a public-access RNA-seq dataset of MDI-induced 3T3-L1 pre-adipocyte to study the role of autophagy in the adipocyte differentiation. First, we examine the changes in gene expression and relative exon usage over time of autophagy-related genes-members of the Gene Ontology term autophagy (GO:0006914). Then, we perform a gene set enrichment analysis and visualize the autophagy pathways to exploit the potential changes and modification in differentiating adipocytes compared to pre-adipocytes. The overall workflow is depicted in Figure 1A. Finally, we present the results in an accessible and searchable format and make it available on the web.

## RESULTS

### Adipocyte differentiation markers and analysis quality assessment

The mouse fibroblast cells, 3T3-L1 pre-adipocytes, differentiate into mature adipocytes when treated with the MDI differentiation medium (160 nM insulin, 250 nM dexamethasone, and 0.5 mM 1-methyl-3-isobutylxanthine). Once fully confluent, 3T3-L1 pre-adipocytes undergo a growth arrest followed by cell division and differentiation upon treatment with MDI [6]. Al Adhami *et al.* performed RNA sequencing of 8 samples of MDI-induced 3T3-L1 cells at 4 time points corresponding to these differentiation stages; day 0 (pre-adipocyte proliferation), day 2 (quiescence state), 10 h (clonal expansion) after MDI treatment and day 6 (differentiation) after MDI treatment [7]. To confirm the differentiation of the adipocytes in this dataset, we first examined the expression levels of 3 important adipocyte markers, which are correlated with the maturation of adipocytes as reported previously [8]; *Cebpa*, CCAAT/enhancer binding protein (C/EBP) alpha; *Lpl*, lipoprotein lipase; *Pparg*, peroxisome proliferator activated receptor gamma. The mRNA levels of these three adipocyte differentiation marker genes relatively increased in differentiating cells (day 6 after MDI treatment) compared to proliferating cells (day 0) (Figure 1B). Additionally, several essential lipogenesis markers including *Fasn*, fatty acid synthase, and *Acaca*, acetyl-CoA carboxylase, were highly elevated in differentiating cells (Figure 1C), indicating that adipogenesis-associated lipid biosynthesis is significantly activated.

To ensure the reliability of our analysis, we performed several steps to quality assess and validate the analysis. First, DESeq2 includes a per-gene estimate of dispersion, which is the within-group variability, in the modeling step. Supplementary Figure 1A shows the per-gene estimates, fitted and final estimates of dispersion versus the mean normalized counts for each gene. Second, the multi-dimensional scaling (MDS) analysis based on total (*n* = 19692) gene counts showed a clear separation between experimental groups (Supplementary Figure 1B). Finally, another dataset composed of 6 RNA-seq samples of MDI-induced 3T3-L1 at 2 time points (day 0 and day 7 after MDI treatment), deposited by Duteil *et al.* [9], was obtained and processed using the same pipeline. The average base expression of autophagy-related genes (*n* = 131) from both datasets showed a high correlation (*Pearson’s* coefficient values = 0.87) between these two independent experiments (Supplementary Figure 1C). These suggest that the dataset we used here is suitable for the autophagy-dependent study in adipogenesis.

### Gene expression of autophagy-related genes in adipogenesis

**Figure 2:**
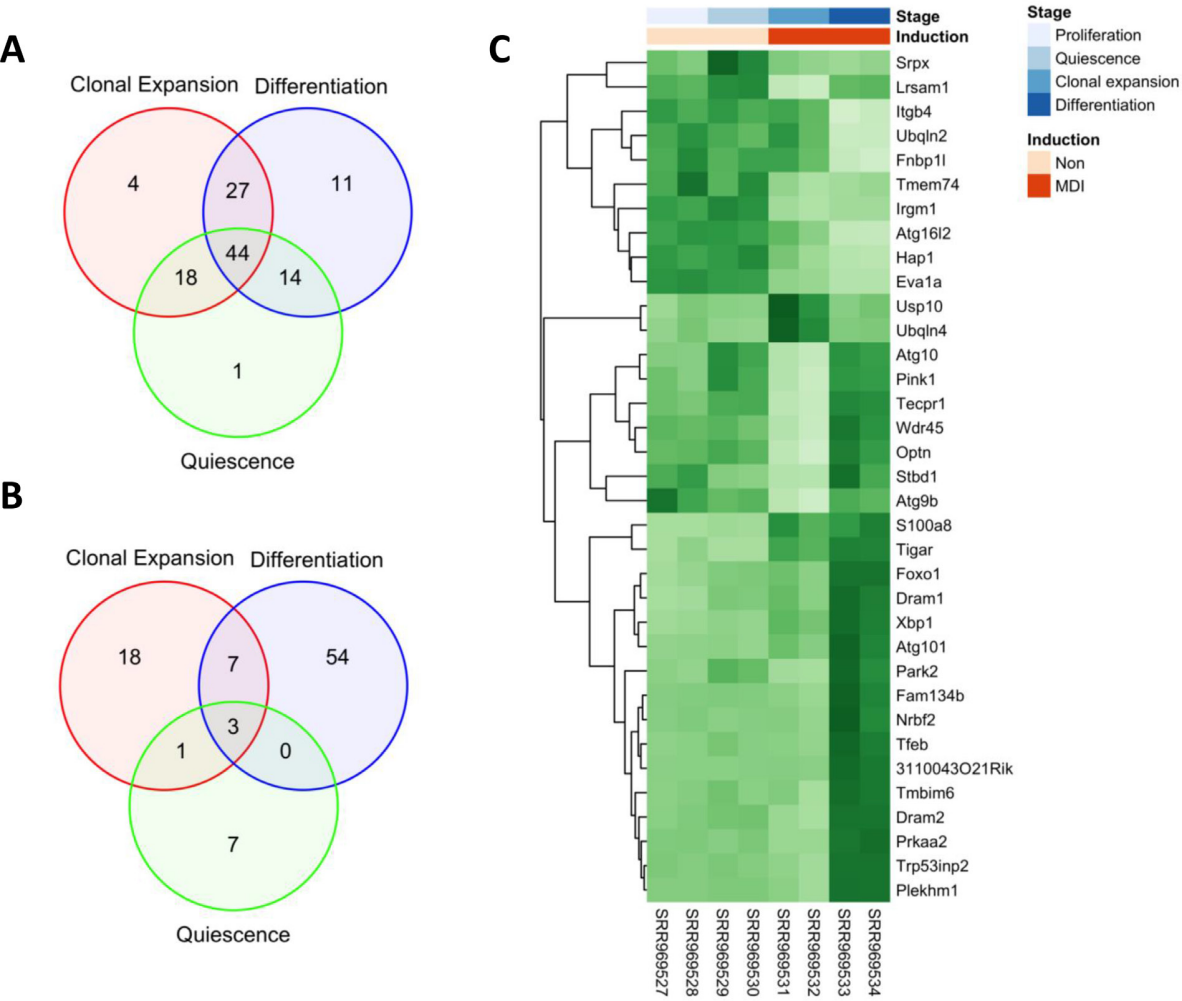
Differential expression of autophagy-related genes and exons during adipocyte differentiation Raw reads of 8 MDI-induced 3T3-L1 RNA-seq samples were aligned to the mm10 reference genome using HISAT2. Aligned reads were counted at feature; gene or exon level using HTSeq. Autophagy-related features were identified using the gene ontology term (autophagy) in the mouse organism annotation package org.Mm.eg.db. Feature counts were used to conduct pairwise comparisons between developmental stages (quiescence, clonal expansion and differentiation) and the control stage (proliferation). (**A**) A summary of DESeq2 differential expression analysis of (*n* = 131) autophagy-related genes. (**B**) A summary of DEXSeq differential exon usage of (*n* = 1777) autophagy-related exons. Venn diagrams show significant features at (adjusted *p*-value < 0.1). (**C**) Log2 gene counts were used to conduct Likelihood Ration Test (LRT) for differential expression among developmental stages; proliferation, quiescence, clonal expansion and differentiation. 35 autophagy-related genes were found significant (adjusted *p*-value < 0.1 and log fold-change > 1) and their scaled counts are shown as a heatmap (*dark green* for high and *light green* for low counts). Rows are labeled by official symbols and columns by the SRA sample identifiers. Sample annotations (time point and induction) are mapped to the corresponding samples by a color key.

In order to examine how overall autophagy genes is regulated with respect to the differentiation stages of 3T3-L1 cells, we systematically analyzed the profiles of autophagy-related gene expression using the workflow model depicted in Figure 1A. First, we conducted a pairwise comparison of the autophagy-related genes expression among the 3 differentiation stages using the proliferating cells as a control, and the numerical summaries are shown in Figure 2A. Of total autophagy-related genes (*n* = 131) identified in the gene ontology term (autophagy), 77 genes were expressed deferentially (adjusted *p*-value < 0.1) between quiescent (day 2) and proliferating cells (day 0), 93 genes between clonal expanding (10 h after MDI treatment) and proliferating cells (day 0), and finally 96 genes between differentiating (day 6 after MDI treatment) and proliferating cells (day 0). Using the Likelihood Ratio Test (LRT), we then tested the different expression pattern across the 4 differentiation stages. Several autophagy-related genes (*n* = 35) were found to be significant (adjusted *p*-value < 0.1 and log fold-change > 1) (Figure 2C).

### The distinct patterns of autophagy-related genes profiles during the adipocyte differentiation

**Figure 3:**
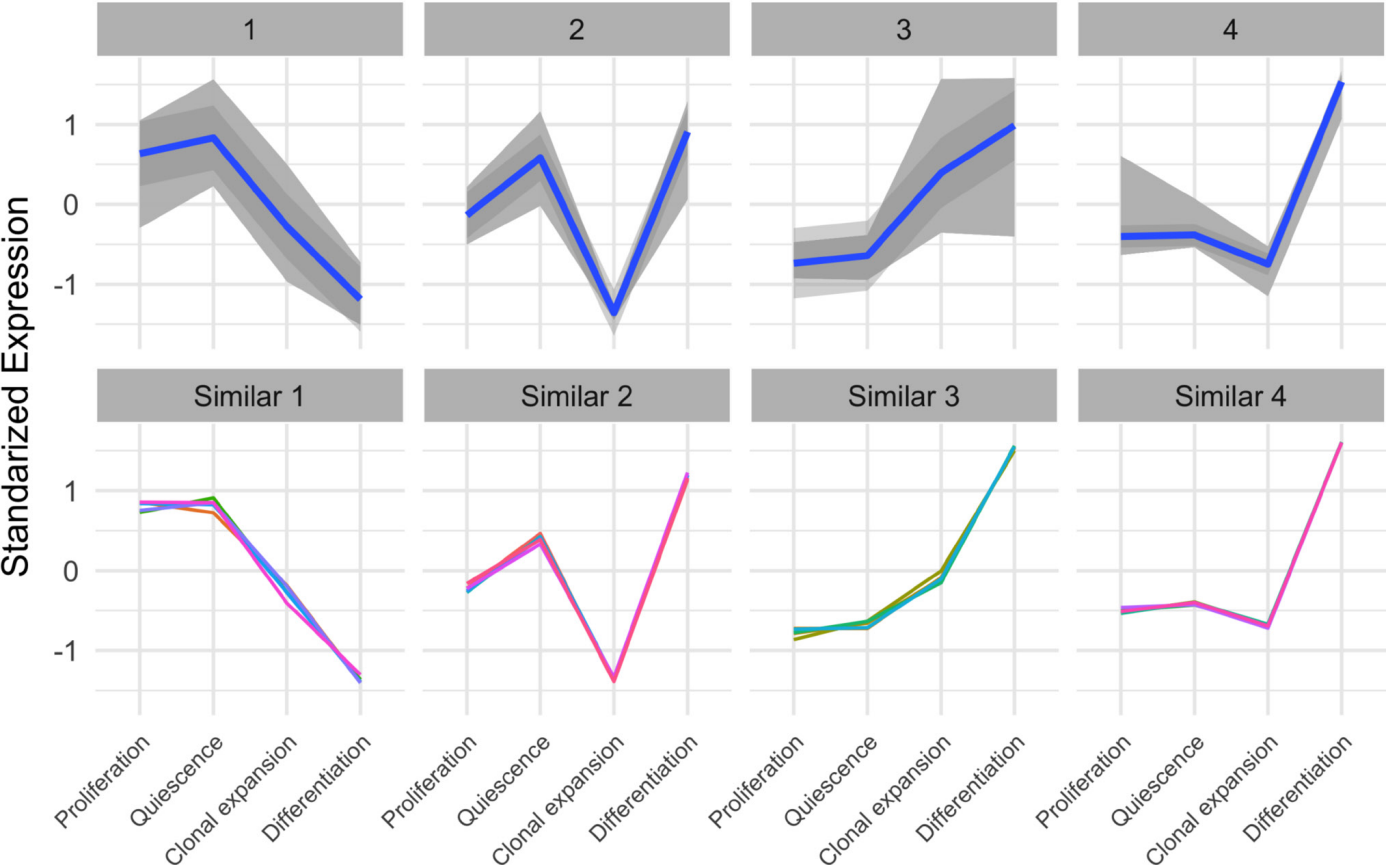
Expression patterns of autophagy-related genes and non-autophagy genes with similar patterns (***top*)** Autophagy-related genes were identified using the gene ontology term (autophagy) in the mouse organism annotation package org.Mm.eg.db., then were clustered using the *Manhattan* distance and the *c*-means algorithm. Each panel represents a distinct cluster (expression profile over time); *gray* color indicates the area spanned by the cluster members, and the *blue* line is the linear trend within the cluster. *y*-axis represents the standardized expression (mean subtracted and divided by the standard deviation). **(*bottom*)** Non-autophagy genes were examined for showing expression patterns similar to the 4 autophagy-related genes’ clusters using the genefinder algorithm in the genefilter package. The 5 closest genes (indicated by different color lines) within each cluster were selected and shown in separate panels. *y*-axis represents the standardized expression (mean subtracted and divided by the standard deviation).

In the Figure 2C, the heatmap suggests that there are 4 distinct patterns (temporal profiles) of autophagy-related gene expression during the course of adipogenesis. To further characterize these patterns, we used the *c*-means algorithm to cluster the autophagy-related genes based on the *Manhattan* distance. Indeed, the resulting 4 clusters (cluster #1∼4) corresponded to the main temporal profiles (Figure 3 top). Except the first cluster, autophagy-related genes exhibited relatively low expression at the proliferating stage (day 0) and a higher expression at the differentiating stage of adipocytes (day 6 after MDI treatment). While autophagy-related genes in cluster 3 showed a gradual increase of expression over time, clusters 2 and 4 showed that expression of some autophagy genes was occasionally decreased at 10 hours after MDI treatment. Genes in the cluster 1 showed a reverse pattern—higher expression at proliferating cells (day 0) and a lower expression at the differentiating cells (day 6 after MDI treatment). The members of each cluster are enumerated by symbol and gene name in Table 1.

**Table 1:** Autophagy-related genes with distinct expression patterns

Cluster	Symbol	Name
1	*Irgm1*	immunity-related GTPase family M member 1
1	*Srpx*	sushi-repeat-containing protein
1	*Ubqln2*	ubiquilin 2
1	*Atg16l2*	autophagy related 16-like 2 (S. cerevisiae)
1	*Fnbp1l*	formin binding protein 1-like
1	*Eva1a*	eva-1 homolog A (C. elegans)
2	*Wdr45*	WD repeat domain 45
2	*Atg10*	autophagy related 10
2	*Pink1*	PTEN induced putative kinase 1
2	*Tecpr1*	tectonin beta-propeller repeat containing 1
2	*Optn*	optineurin
2	*Lrsam1*	leucine rich repeat and sterile alpha motif containing 1
3	*S100a8*	S100 calcium binding protein A8 (calgranulin A)
3	*Usp10*	ubiquitin specific peptidase 10
3	*Xbp1*	X-box binding protein 1
3	*Foxo1*	forkhead box O1
3	*Atg101*	autophagy related 101
3	*Dram1*	DNA-damage regulated autophagy modulator 1
3	*Ubqln4*	ubiquilin 4
3	*Tigar*	Trp53 induced glycolysis repulatory phosphatase
4	*Tfeb*	transcription factor EB
4	*Park2*	Parkinson disease (autosomal recessive, juvenile) 2, parkin
4	*Stbd1*	starch binding domain 1
4	*Fam134b*	family with sequence similarity 134, member B
4	*Dram2*	DNA-damage regulated autophagy modulator 2
4	*Trp53inp2*	transformation related protein 53 inducible nuclear protein 2
4	*3110043O21Rik*	RIKEN cDNA 3110043O21 gene
4	*Prkaa2*	protein kinase, AMP-activated, alpha 2 catalytic subunit
4	*Tmbim6*	transmembrane BAX inhibitor motif containing 6
4	*Plekhm1*	pleckstrin homology domain containing, family M (with RUN domain) member 1
4	*Nrbf2*	nuclear receptor binding factor 2

Next, we used an algorithm to find other non-autophagy genes, not identified as autophagy-related genes in the gene ontology term (autophagy), with similar expression patterns. We first identified the clusters’ mediods—a gene with the most typical pattern to a certain cluster—for each of the above described clusters and used the genefinder algorithm in the genefilter package to find the 5 closest genes to each of the clusters’ mediods. We showed that the expression patterns of the closer 20 genes (4 groups: 5 genes in each group) to the previously identified clusters (Figure 3 bottom) and listed their symbols and gene names in Table 2.

**Table 2:** Non-autophagy genes with similar expression patterns

Cluster	Symbol	Name
Similar 1	*Sfxn3*	sideroflexin 3
Similar 1	*Plxnb2*	plexin B2
Similar 1	*Map4*	microtubule-associated protein 4
Similar 1	*Syde1*	synapse defective 1, Rho GTPase, homolog 1 (C. elegans)
Similar 1	*Arpin*	actin-related protein 2/3 complex inhibitor
Similar 2	*Ralgps1*	Ral GEF with PH domain and SH3 binding motif 1
Similar 2	*Afap1l2*	actin filament associated protein 1-like 2
Similar 2	*Pick1*	protein interacting with C kinase 1
Similar 2	*Ypel2*	yippee-like 2 (Drosophila)
Similar 2	*Slc29a3*	solute carrier family 29 (nucleoside transporters), member 3
Similar 3	*Dnaja3*	DnaJ heat shock protein family (Hsp40) member A3
Similar 3	*Plekhf2*	pleckstrin homology domain containing, family F (with FYVE domain) member 2
Similar 3	*Asgr2*	asialoglycoprotein receptor 2
Similar 3	*Kcnn1*	potassium intermediate/small conductance calcium-activated channel, subfamily N, member 1
Similar 3	*Pex10*	peroxisomal biogenesis factor 10
Similar 4	*Mccc2*	methylcrotonoyl-Coenzyme A carboxylase 2 (beta)
Similar 4	*Slc22a23*	solute carrier family 22, member 23
Similar 4	*Pex19*	peroxisomal biogenesis factor 19
Similar 4	*Lonp2*	lon peptidase 2, peroxisomal
Similar 4	*Tmem177*	transmembrane protein 177

**Figure 4:**
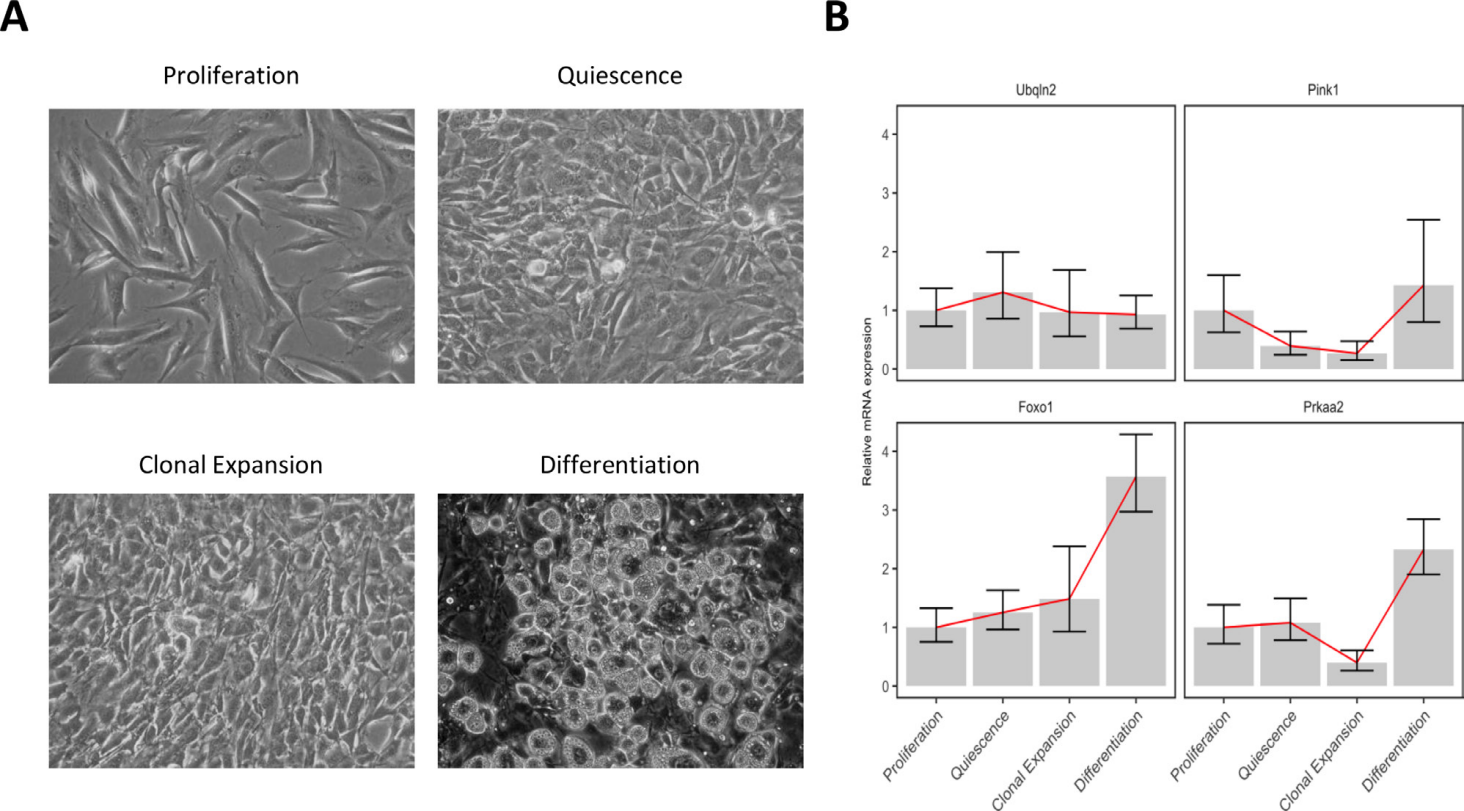
3T3-L1 adipocyte differentiation and gene expression validation 3T3-L1 pre-adipocytes were cultured in initiated and induced by the MDI differentiation mixture. Total RNAs were collected at 4 different time points and real-time quantitative PCR was used to quantify the mRNA relative expression. (**A**) Microscopic pictures of the 3T3-L1 cells at different stages: proliferation (day -2), quiescence (day 0), clonal expansion (10 hours after MDI indection), and differentiation (6 days after MDI induction). (**B**) mRNA expression of 4 genes at the same differentiation stages was normalized to the control gene 18S and its relative expression was determined by comparing to the first stage (proliferation). *Foxo1*, forkhead box O; *Pink1*, PTEN induced putative kinase 1; *Prkaa2*, protein kinase, AMP-activated, alpha 2 catalytic subunit; *Ubqln2*, ubiquilin 2. Lines (*red*) indicate the trend of the mean values of each gene expression at the differentiation stages.

In order to validate the expression pattern of genes belong to each autophagy cluster, we performed the differentiation of 3T3-L1 pre-adipocytes in the MDI differentiation medium as described in “Materials and Methods” and collected total RNAs from cells with four different stages; proliferation (day -2), quiescence (day 0), clonal expansion (10 h after MDI induction) and differentiation (6 days after MDI induction) shown in Figure 4A. The quantitative PCR data of some representative genes: *Ubqln2* (cluster #1), *Pink1* (cluster #2), *Foxo1* (cluster #3), and *Prkaa2* (cluster #4), showed that the relative expression pattern of each gene at differentiation stages well agreed with the depicted patterns by members of the four autophagy clusters shown in Figure 3 and Table 1 (Figure 4B).

### Enrichment of autophagy gene sets during the adipocyte differentiation

**Figure 5:**
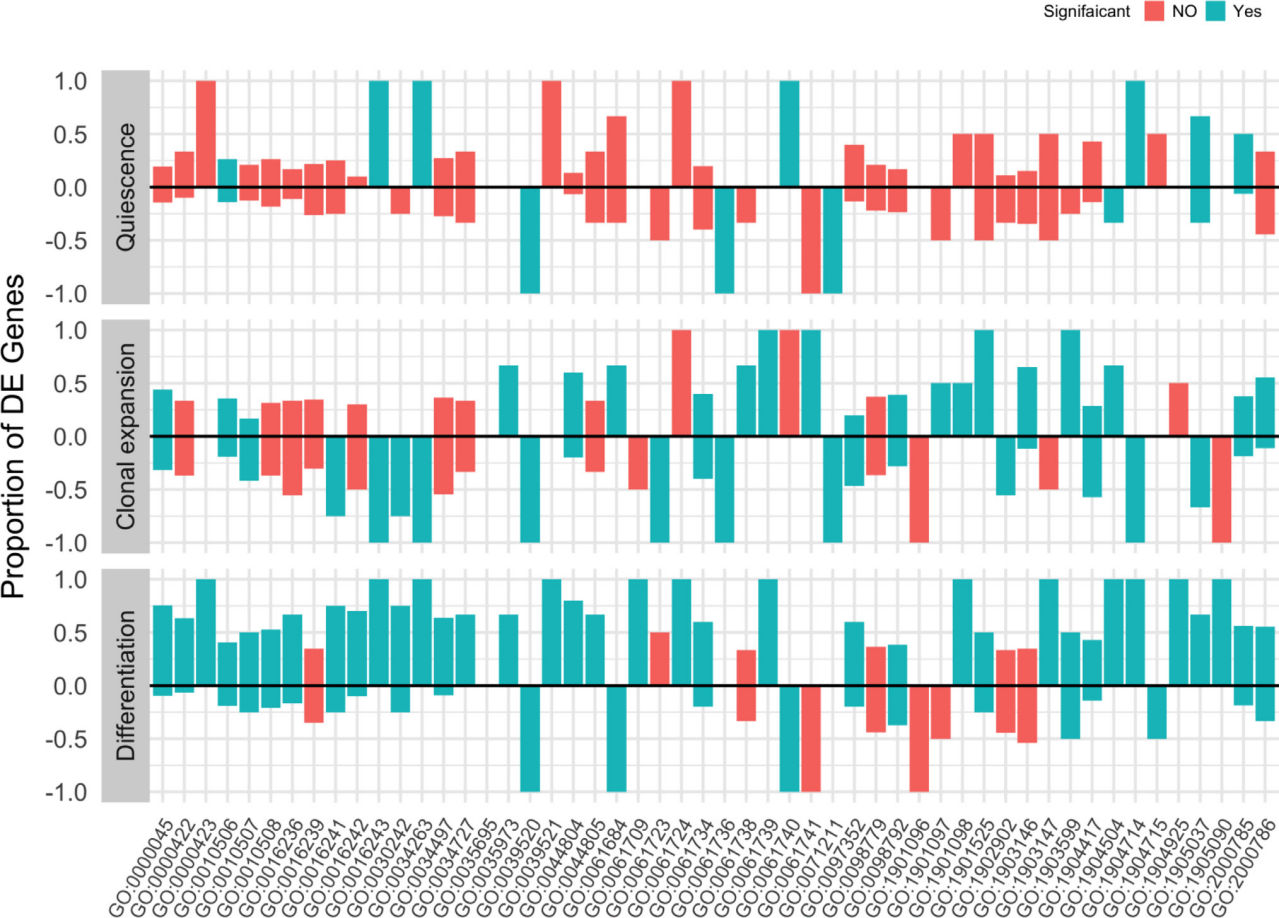
Enrichment of autophagy gene sets during adipocyte differentiation Autophagy-related genes were identified using the gene ontology term (autophagy) in the mouse organism annotation package org.Mm.eg.db. Package limma were used to test for the gene set enrichment by applying the rotation test to the fragment per million (fpm) matrix and the comparisons between proliferating cells (day 0) and quiescent cells (day 2), clonal expanding cells (10 h after MDI treatment) and differentiating cells (day 6 after MDI treatment). Bars represent the proportion of genes in a gene set (labeled by GO ids) that are deferentially expressed at a certain comparison. Color indicates whether the overall gene set enrichment is significant (false discovery rate < 0.1) (*green,* yes or *red,* not).

**Figure 6:**
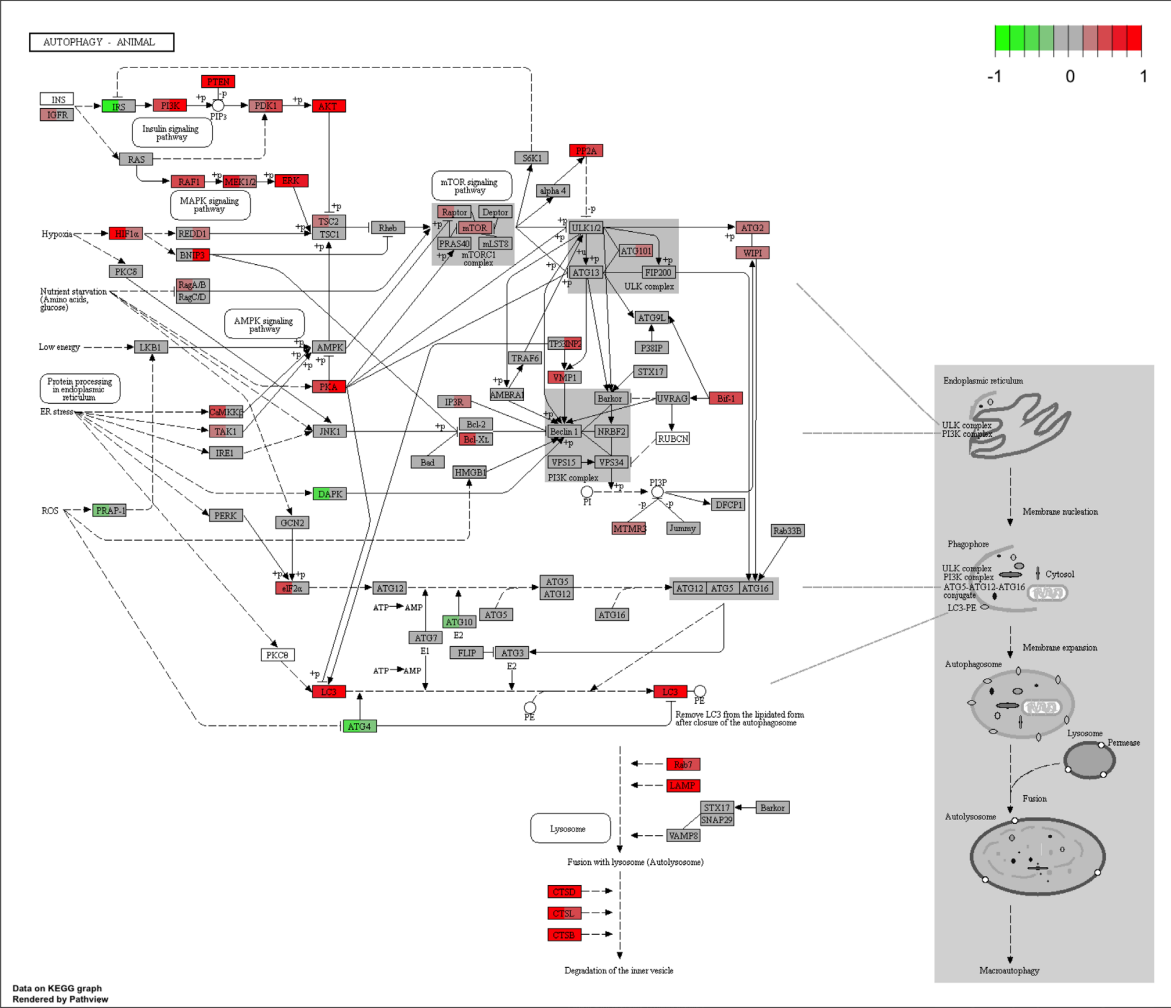
Autophagy KEGG pathway map in proliferating pre-adipocytes and differentiating adipocytes Mouse autophagy pathway (04140) graph was obtained from the KEGG pathway database using pathview package. Scaled fragment per million (fpm) of genes were used to map the expression levels (*red* for high and *green* for low) to the graph. Each node is divided to two parts; *left*, proliferating pre-adipocytes (day 0) and *right*, differentiating adipocyte cells (day 6 after MDI treatment).

To further understand the ongoing regulation of autophagy at the gene set level during adipogenesis, we first identified the autophagy offspring (sub-categories) gene sets in the gene ontology. Then, we used the gene count/fragment per million (fpm) to test for their differential enrichment during courses of adipocyte differentiation. Few autophagy gene sets showed significant enrichment (False Discovery Rate < 0.1) between quiescent and proliferating cells. However, a high number of autophagy gene sets were significantly enriched between adipocyte differentiating cells and proliferating control cells due to a largely increased proportion of deferentially expressed genes between the two stages (Figure 5). We present the proportion of deferentially expressed genes for each autophagy gene set at three different conditions (quiescence, clonal expansion, differentiation) compared to the control condition (proliferation) in Figure 5 and listed the corresponding false discovery rates in Table 3. To better visualize the changes in the autophagy process during the adipocyte differentiation, we mapped the standardized average gene counts of two conditions (left, proliferation and right, differentiation) as color values to the mouse KEGG pathway for autophagy (Figure 6).

**Table 3:** Enrichment of autophagy gene sets during adipocyte differentiation

GO ID	Term	Quiescence	Clonal Expansion	Differentiation
GO:0000045	autophagosome assembly	0.86	0.01	<0.01
GO:0000422	mitophagy	0.58	0.66	<0.01
GO:0000423	macromitophagy	0.20	0.43	<0.01
GO:0010506	regulation of autophagy	0.02	0.01	<0.01
GO:0010507	negative regulation of autophagy	0.52	0.03	<0.01
GO:0010508	positive regulation of autophagy	0.20	0.44	<0.01
GO:0016236	macroautophagy	0.92	0.62	<0.01
GO:0016239	positive regulation of macroautophagy	0.89	0.44	0.65
GO:0016241	regulation of macroautophagy	0.91	0.02	<0.01
GO:0016242	negative regulation of macroautophagy	0.70	0.69	<0.01
GO:0016243	regulation of autophagosome size	0.07	0.10	0.01
GO:0030242	pexophagy	0.83	0.06	0.01
GO:0034263	positive regulation of autophagy in response to ER overload	0.05	0.09	<0.01
GO:0034497	protein localization to pre-autophagosomal structure	0.95	0.13	<0.01
GO:0034727	piecemeal microautophagy of nucleus	0.89	0.31	<0.01
GO:0035695	mitophagy by induced vacuole formation	0.41	0.33	0.28
GO:0035973	aggrephagy	0.38	<0.01	<0.01
GO:0039520	induction by virus of host autophagy	0.06	0.01	<0.01
GO:0039521	suppression by virus of host autophagy	0.24	0.60	<0.01
GO:0044804	nucleophagy	1.00	<0.01	<0.01
GO:0044805	late nucleophagy	0.90	0.31	<0.01
GO:0061684	chaperone-mediated autophagy	0.22	0.02	0.04
GO:0061709	reticulophagy	0.92	0.19	<0.01
GO:0061723	glycophagy	0.53	0.04	0.12
GO:0061724	lipophagy	0.12	0.14	0.02
GO:0061734	parkin-mediated stimulation of mitophagy in response to mitochondrial depolarization	0.40	0.01	<0.01
GO:0061736	engulfment of target by autophagosome	0.05	<0.01	0.39
GO:0061738	late endosomal microautophagy	0.45	<0.01	0.14
GO:0061739	protein lipidation involved in autophagosome assembly	0.66	0.01	0.01
GO:0061740	protein targeting to lysosome involved in chaperone-mediated autophagy	0.06	0.19	0.05
GO:0061741	chaperone-mediated protein transport involved in chaperone-mediated autophagy	0.22	<0.01	0.12
GO:0071211	protein targeting to vacuole involved in autophagy	0.04	<0.01	0.41
GO:0097352	autophagosome maturation	0.10	0.05	<0.01
GO:0098779	positive regulation of macromitophagy in response to mitochondrial depolarization	0.95	0.97	0.35
GO:0098792	xenophagy	0.59	0.02	<0.01
GO:1901096	regulation of autophagosome maturation	0.37	0.10	0.20
GO:1901097	negative regulation of autophagosome maturation	0.42	<0.01	0.57
GO:1901098	positive regulation of autophagosome maturation	0.12	0.09	<0.01
GO:1901525	negative regulation of macromitophagy	0.72	0.01	<0.01
GO:1902902	negative regulation of autophagosome assembly	0.56	0.06	0.31
GO:1903146	regulation of mitophagy	0.28	<0.01	0.55
GO:1903147	negative regulation of mitophagy	0.78	0.17	<0.01
GO:1903599	positive regulation of mitophagy	0.22	<0.01	<0.01
GO:1904417	positive regulation of xenophagy	0.24	0.01	<0.01
GO:1904504	positive regulation of lipophagy	0.02	<0.01	<0.01
GO:1904714	regulation of chaperone-mediated autophagy	0.04	0.01	<0.01
GO:1904715	negative regulation of chaperone-mediated autophagy	0.58	0.20	0.07
GO:1904925	positive regulation of mitophagy in response to mitochondrial depolarization	0.94	0.22	0.01
GO:1905037	autophagosome organization	0.08	0.05	<0.01
GO:1905090	negative regulation of parkin-mediated stimulation of mitophagy in response to mitochondrial depolarization	0.56	0.19	<0.01
GO:2000785	regulation of autophagosome assembly	0.06	0.04	<0.01
GO:2000786	positive regulation of autophagosome assembly	0.47	0.01	0.01

False discovery rates of rotation tests of autophagy gene ontology offspring gene sets in three differentiation stages compared to control

Similarly, we identified the signaling pathways in the KEGG database that share at least one of their members with the autophagy regulation pathway. After testing for the enrichment of the pathways using the fragment per million gene counts, we listed the significant pathways in the later three differentiation stages (quiescence, clonal expansion, differentiation) compared to the earliest proliferation stage in Table 4. The mTOR, Jak-STAT, insulin and adipocytokine signaling pathways were highly enriched between differentiating and non-differentiating (proliferating) cells (Supplementary Figure 2), suggesting that the role of these signaling pathways in adipogenesis is associated, at least in part, with regulating autophagy.

**Table 4:** Enrichment of KEGG Pathways sharing autophagy related genes

Pathway	Quiescence	Clonal Expansion	Differentiation
Malaria	0.08	0.02	0.05
mTOR signaling pathway	0.09	0.09	<0.01
Regulation of actin cytoskeleton	0.11	0.27	<0.01
Maturity onset diabetes of the young	0.12	0.01	0.01
Progesterone-mediated oocyte maturation	0.14	0.08	0.01
Oocyte meiosis	0.15	0.85	0.05
Cytokine-cytokine receptor interaction	0.19	<0.01	0.37
Amoebiasis	0.20	0.06	0.63
Cytosolic DNA-sensing pathway	0.29	0.01	0.03
Insulin signaling pathway	0.31	0.67	<0.01
Antigen processing and presentation	0.36	0.10	0.21
Autoimmune thyroid disease	0.36	0.35	<0.01
Regulation of autophagy	0.37	0.01	<0.01
Allograft rejection	0.41	0.47	0.08
Osteoclast differentiation	0.51	0.37	0.09
Proteasome	0.51	0.03	0.06
Prostate cancer	0.52	0.48	0.72
Phagosome	0.55	0.35	0.09
Metabolic pathways	0.62	0.03	<0.01
RIG-I-like receptor signaling pathway	0.64	0.46	0.01
Graft-versus-host disease	0.66	0.85	<0.01
Aldosterone-regulated sodium reabsorption	0.66	0.01	<0.01
Jak-STAT signaling pathway	0.67	0.11	<0.01
Systemic lupus erythematosus	0.75	0.59	0.20
Toxoplasmosis	0.75	0.95	0.02
Leishmaniasis	0.75	0.19	0.06
Type II diabetes mellitus	0.75	0.38	<0.01
Natural killer cell mediated cytotoxicity	0.75	0.07	0.25
Hypertrophic cardiomyopathy (HCM)	0.76	0.01	<0.01
African trypanosomiasis	0.76	0.46	0.23
Rheumatoid arthritis	0.76	0.06	0.01
T cell receptor signaling pathway	0.76	0.23	0.05
TGF-beta signaling pathway	0.81	0.01	0.03
Toll-like receptor signaling pathway	0.87	0.03	0.06
Chagas disease (American trypanosomiasis)	0.88	0.83	0.05
Hepatitis C	0.90	0.08	0.49
Phosphatidylinositol signaling system	0.90	0.10	0.53
Type I diabetes mellitus	0.96	0.50	0.30
Inositol phosphate metabolism	0.97	0.65	0.02
Adipocytokine signaling pathway	0.99	<0.01	<0.01

False discovery rates of rotation tests of KEGG pathways that shares one or more of autophagy-related genes in three differentiation stages compared to control

### Differential exon usage of autophagy genes in adipogenesis

**Figure 7:**
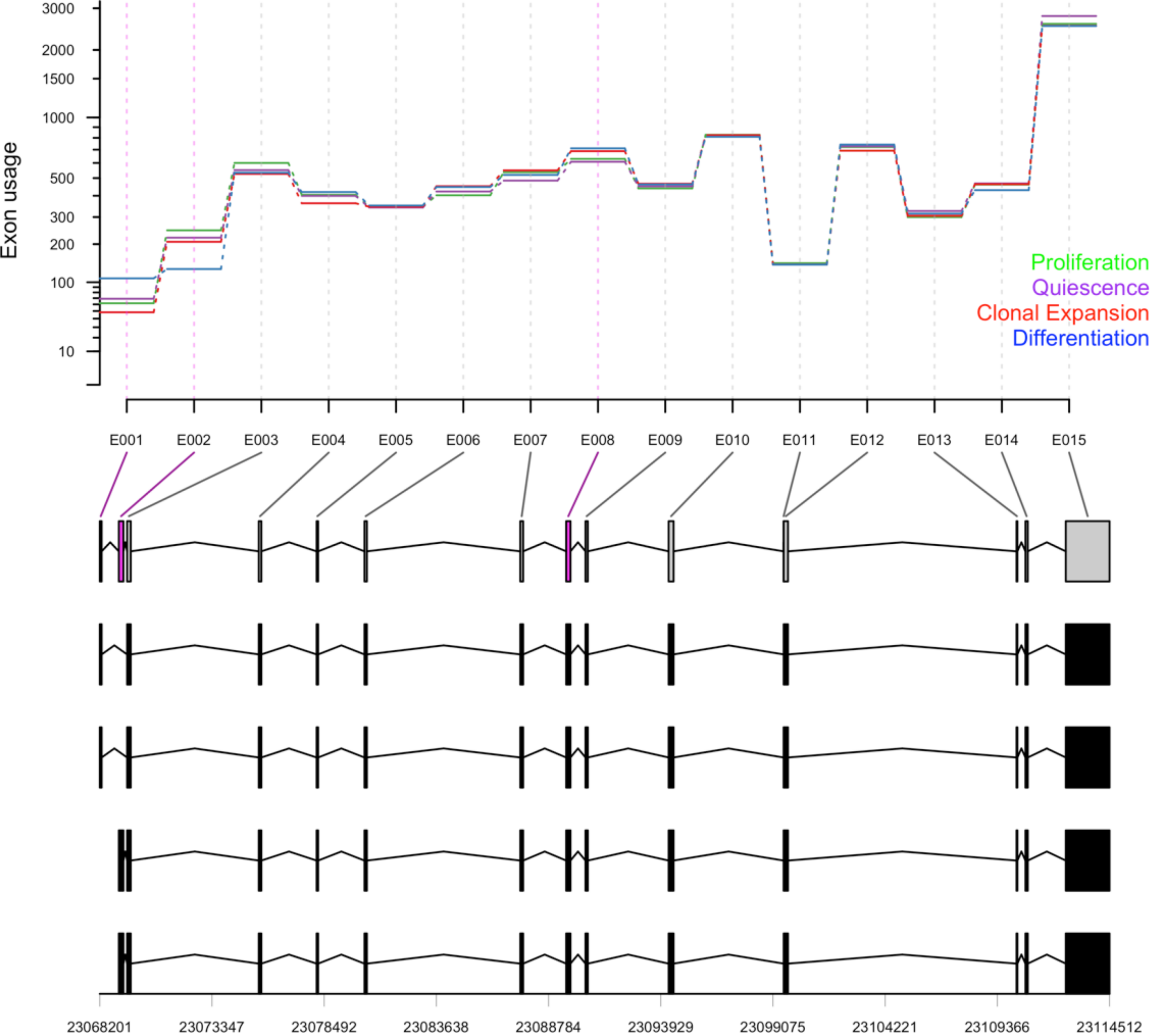
Differential exon usage of *Acbd5* Counts of total 16073 exons in 8 samples were used to test for differential usage using DEXSeq for the pairwise comparison between proliferating samples (day 0) and quiescence (day 2), clonal expansion (10 h after MDI treatment) and differentiating samples (day 6 after MDI treatment). Exon usage values of 19 exons of *Acbd5* at different differentiation stages (*green*, proliferation; *purple*, quiescence; *red*, clonal expansion; *blue*, differentiation) are shown with their corresponding transcripts and genomic ranges at the bottom.

In addition to the changes of the expression level of genes involved in the cellular process, relative usage of certain exons and the abundance of certain transcripts is an additional potential mechanism for regulation and modification of autophagy during the adipocyte differentiation. We tested for the differential exon usage in the eight samples of 3T3-L1 cells used in this study and identified potential alternative splicing events. A few of these events were identified in quiescent cells and more events at the clonal expansion and differentiating cells compared to the control proliferating cells. We summarized the differential exon usage events among autophagy-related exons (*n* = 1777) in Figure 2B. In addition, we presented the relative usage of 15 exons of *Acbd5* (acyl-CoA binding domain containing 5), a gene known to function in the transport and distribution of long chain acyl-Coenzyme A in cells, during the adipocyte differentiation in Figure 7. Exons 1, 2 and 8 showed significant relative usage over time (adjusted *p*-value < 0.1). Exon 2 is only a part of 2 transcripts (*bottom*), which indicates a potential splicing event. In other words, transcripts containing exon 2 were less expressed in differentiating cells compared to earlier stages and probably the first two transcripts were more abundant.

## DISCUSSION

Autophagy is an essential process during the pre-adipocyte 3T3-L1 differentiation into mature adipocytes. According to previous studies, inhibition of autophagy by knockdown of *Atg5* or *Atg7* genes in 3T3-L1 pre-adipocytes prevents the adipogenesis and the formation of lipid droplets. Also, autophagy inhibition causes a significant decrease in adipocyte differentiation markers, such as CCAAT/enhancer-binding protein-alpha (*CEBPa*) and peroxisome proliferator activated receptor-gamma (*PPARg*) [10]. In this report, we examined the differential expression of autophagy-related genes at the transcriptional level during the adipocyte differentiation using public-access RNA-seq datasets. Our analysis reveals that the expression of *Atg5* and *Atg7* genes is maintained at a high level during the course of differentiation with respect to the maturation stage when 3T3-L1 pre-adipocytes are induced in the differentiation medium (data not shown). Although we cannot quantify the lipid contents in this study, we show the relatively high expression of a number of protein markers that are highly correlated with lipogenesis in adipose tissue in Figure 1C [11].

Other cells than adipocytes also have the ability to store different amounts of lipids, for instance, hepatocytes and neurons; in some pathological conditions [10, 12]. In particular, hepatocytes use autophagy to generate free lipids from lipid droplets for energy metabolism in a specialized lipid degradation process called lipophagy [10]. In addition to the role of autophagy in lipid droplet formation in adipogenesis, autophagy also takes a significant part in the lipolytic pathway. The gene ontology term lipophagy represented by one gene member, RAS oncogene family (*Rab7*), appears to be slightly activated in differentiating adipocyte compared to the control pre-adipocytes. In addition, adrenergic receptor, beta 2 (*Adrb2*), a member of the term positive regulation of lipophagy is significantly up-regulated in the same comparison. However, how these two lipophagy-associated genes at the gene ontology term can function in the canonical autophagy process still remains to be demonstrated.

The classic ability of autophagy to recycle the cellular components is particularly important during the adipocyte differentiation. Autophagy targets different intracellular organelles, cytoplasmic regions and protein aggregates. This in turn allows adipocytes to store large amounts of lipids and to gain a distinct morphology. Additionally, the nutrients acquired by the autophagy-dependent degradation in adipogenesis can be good sources for adipocyte growth and lipid biosynthesis. The gene set enrichment analysis reveals activation of autophagy gene subsets responding to distinct intracellular targets; mitophagy (mitochondria), nucleophagy (nucleus), reticulophgay (endoplasmic reticulum), pexophagy (peroxisomes), xenophagy (cytoplasmic regions) and aggrephagy (protein aggregates). Moreover, mitophagy-specific genes such as *Pink1*, or *Park2* [13–15] are differently expressed at different time points during the adipocyte differentiation. Also, *Fam134b* gene is specifically involved in the selective removal of endoplasmic reticulum under certain conditions [16, 17]. *Optn* gene also plays a critical role in degradation of some cytoplasmic regions via xenophagy [18]. Indeed, we showed that the expression of these two genes was regulated in a developmental stage-specific manner during adipocyte differentiation.

Given the role of autophagy in adipocyte differentiation for lipid droplet formation and recycling the cellular components, we might also expect some significant quantitative changes in the process or in its associated regulatory pathways. It is not clear whether the quantification of autophagosomes at a certain time point without blocking the autophagy flux is possible. Nonetheless, transcript levels of both microtubule associated protein 1 light chain 3 (*maplc3a*) and autophagy-related 5 (*Atg5*) are highly correlated to autophagosome formation. As shown in (Figure 5 and Table 3), we observed a significant up-regulation of members of the autophagosome assembly genes (31% and 75% up-regulation at 10 h and 6 days after induction, respectively; adjusted *p*-value < .01) Consequently, we also observe a significant enrichment of autophagy-related gene ontology terms; namely protein localization to pre-autophagosomal structure and protein lipidation involved in autophagosome assembly. Taken together, we can infer a quantitative increase of autophagy during the adipocyte differentiation based on increased transcription levels of *Map1lc3a* and other genes involved in its localization and lipidation.

Sub-grouping process of autophagy to several offspring gene sets in the gene ontology can give the detailed information to understand the role of autophagy in adipogenesis at the gene set level. Based on only mRNA levels obtained from public-access RNA-seq data, we indeed found a significant enrichment of many autophagy gene sets during the course of adipocyte differentiation although there still would be difficult to understand how each autophagy gene set is exactly related to among different types of autophagy.

Detailed knowledge of relative gene expression levels of key genes in autophagy-related gene sets and their overall enrichment over time could be used to emphasis certain aspects of the involvement of autophagy in adipocyte differentiation. Basal autophagy is activated when pre-adipocytes respond to the differentiation stimulus. Organelle-specific autophagy is probably employed to achieve the structural modification and meet the metabolic demands in mature adipocytes. Quantitative and regulatory changes are indeed required to govern the autophagy response during differentiation. Not only autophagy-related gene sets such as autophagosome assembly and signaling pathways such as mTOR and Jak-STAT but also adipogenic pathways like insulin and adipocytokine signaling show a significant enrichment at the differentiation stage of adipocytes compared to the control stage.

Much of the regulation of autophagy in adipogenesis is probably happening at protein and protein modification levels, and wouldn’t be easily captured by the changes at the mRNA level. Using parallel datasets from proteomic, RNA-seq or transcription factors ChIP-seq studies is one way to overcome the limitation of general protein functional approaches shown in many other studies [2, 3, 10]. Also, assessing the lipid contents or the autophagosome formation in a certain condition is not feasible by sequencing data alone. Therefore, integrating datasets of varies measurement technologies is a plausible future application of these findings.

In conclusion, the systematic analysis using public-access databases such as microarray or RNA-seq can provide new valuable information to collectively understand a specific cellular pathway. Here, we show that expression of many autophagy-related genes are highly up-regulated at the differentiation stages of adipocytes in accordance with previous reports, and autophagy gene sets defined in the gene ontology are enriched among the developmental stages, further confirming the critical role of autophagy in adipogenesis.

## MATERIALS AND METHODS

### Raw data and processing

The dataset (Al Adhami *et al.*) consists of 8 samples (two of each time point) of 3T3-L1 pre-adipocytes treated with/without MDI (160 nM insulin, 250 nM dexamethasone, and 0.5 mM 1-methyl-3-isobutylxanthine) at four time points; day 0 (pre-adipocyte proliferation), day 2 (quiescence state) and 10 h (clonal expansion) after MDI treatment, day 6 (differentiation) after MDI treatment. The protocol for generating the RNA libraries and sequencing is described at [7]. Raw reads for 8 samples were obtained from ENA (PRJNA218101). Reads were aligned to the mouse UCSC (mm10) reference genome using the splicing aligner HISAT2 [19]. Aligned reads were counted using HTSeq [20] and summarized at gene and exon level guided by the corresponding annotation data from the UCSC mm10. A validation dataset (Duteil *et al*) consists of 6 samples (three of each time point) of 3T3-L1 cells at two time points day 0 and day 7 after MDI treatment. The protocol for generating the RNA libraries and sequencing is described at [9]. Raw data were obtained from ENA (PRJNA219405) and processed by the same above mentioned pipeline to summarize counts at gene level.

### Differential expression and exon usage

Pairwise comparison between control (proliferation) and 3 conditions (quiescence, clonal expansion and differentiation) were conducted by applying Wald test of the negative binomial distribution to the log2 gene counts using DESeq2 [21]. Likelihood Ratio Test (LRT) was also applied using DESeq2 to test for differential expression across all conditions. DEXSeq was used to test for the differential exon usage [22]. *p*-values were adjusted for multiple testing using BH and the cutoff 0.1 was chosen. DESeq2 and DEXSeq estimate a size factor, that is the median of the ratios of the observed counts, one value for each sample as a normalization factor to correct for potential sequencing depth and other biases to make gene measurements form different samples comparable. We limited the results to the group of genes of known function or relation to autophagy as identified by the gene ontology term autophagy (GO:0006914) and referred to them as autophagy-related genes.

### Clustering and pattern finding

C-means algorithm in the e1071 package was used to cluster the genes into distinct temporal profile. C-mean is a fuzzy version of the *k*-means algorithm [23]. Package genefilter was used to find genes with similar expression pattern to the clusters’ mediods—genes with the most typical pattern to identified cluster [24]. For a certain cluster, genefinder calculates the *Euclidean* distance of all genes to the a mediod, ranks genes by their distance and chooses the *n* closest genes to the mediod.

### Gene set enrichment analysis

Gene ontology (GO) was used to identify the autophagy gene sets and related genes [25]. KEGG pathways were used to identify signaling pathways that share one or more of the autophagy related genes [26]. The mouse organism annotation package (org.Mm.eg.db) was used to retrieve the relative annotations [27]. Gene counts were first transformed to the fragment per million and using package limma a rotation test for the gene set and pathway enrichment was applied to the comparison between control (proliferation, day 0) and 3 conditions (quiescence, day 2; clonal expansion, 10 h after treatment; differentiation, day 6 after treatment) [28]. *p*-values were adjusted for multiple testing using False Discovery Rate (FDR) and the cutoff 0.1.

### Cell culture, MDI induction, and qPCR

3T3-L1 pre-adipocytes were cultured and maintained in Dulbecco’s Modified Eagle Medium (DMEM) supplemented with 10% calf serum (Gibco, Carlsbad, CA, USA) at 37° C in a humidified atmosphere of 5% CO_2_. At 100% confluence, cells were transferred to a fresh medium and incubated for 2 days. Arrested cell were then moved to fresh DMEM supplemented with 10% FBS and the induction mixture MDI (160 nM insulin, 250 nM dexamethasone and 0.5 nM 1-methyl-3-isobutylxanthine). The medium was then changed twice at 2 days’ interval to DMEM + 10% FBS and in addition to 160 nM at the first change only. Cells were harvested at 4 time points corresponding to the 4 differentiation stages; proliferation (day -2), quiescence (day 0), clonal expansion (10 hours after MDI induction), and differentiation (6 days after MDI induction). Total RNAs were purified using TRIzol and assessed by measuring the 260/230 diffraction ratios to ensure the quality of RNA samples and to calculate the proper amount for the qPCR quantification.

One-step real-time quantitative PCR (QuantiNova SYBR Green PCR kit, QIAGEN, Rotor-Gene Q) was used to amplify and quantify the mRNA levels in three independent samples from the 4 time points. The primers used for qPCR were as follows; m*Ubqln2* (forward-TTGAGCTGTTCCAGTTGCTG, reverse- ACCCAACCAGCAGTTCATTC), m*Pink1* (forward- AGTGTCCAGTGGGTCAGACA, reverse- CTGATCGAGGAGAAGCAGG), m*Foxo1* (forward- TGCTGTGAAGGGACAGATTG, reverse- GAGTGGATGGTGAAGAGCGT), and m*Prkaa2* (forward- ACTGCCACTTTATGGCCTGT, reverse- GATCGGACACTACGTCCTGG), and m*18S* (forward-ACCGCAGCTAGGAATAATGGA, reverse- GCCTCAGTTCCGAAAACCA). The *pcr* package was used to apply the comparative CT method on the data. CT values were normalized to that of the control 18S and calibrated to the first time point (proliferation stage).

### Software environment and reproducibility

The data analysis and visualization were mainly done in an R environment. A fully reproducible version of this manuscript is available at (https://github.com/MahShaaban/3T3-L1) along with the scripts that were used to process and analyze the data.

## SUPPLEMENTARY MATERIALS FIGURES


