# Supplementary material for: Systematic characterization of autophagy-related genes during the adipocyte differentiation using public-access data
Source: Oncotarget. 2018 Feb 15;9(21):15526–41. doi: 10.18632/oncotarget.24506 (PMC5884645; doi:10.18632/oncotarget.24506)
Supplement: Supplementary file 1 [file oncotarget-09-15526-s001.pdf]

Systematic characterization of autophagy-related genes during the adipocyte differentiation using public-access data

SUPPLEMENTARY MATERIALS

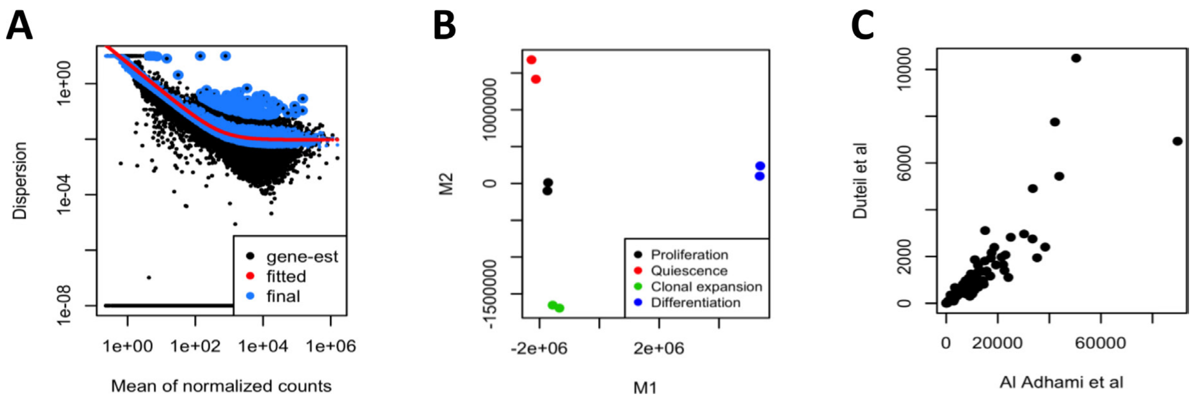

Supplementary Figure 1: Quality assessment, exploration and validation of datasets. (**A**) The estimated genes (*black*), fitted (*red*) and final dispersion (*blue*) data of Al Adhami *et al* [7] calculated by DESeq2 were plotted against the mean normalized counts for numbers of genes across 8 samples. (**B**) Multidimensional Scaling (MDS) was applied to the count matrix of 8 samples and the two dimensions were mapped to the 4 stages of adipocyte differentiation; proliferation (*black*), quiescence (*red*), clonal expansion (*green*) and differentiation (*blue*). (**C**) Six samples of MDI-induced 3T3-L1 at day 0 and day 7 of the other dataset (Duteil *et al* [9]) were processed using the same pipeline and tested for differential expression and used for validating the main findings. Log fold-changes of 35 autophagy-related genes from the corresponding comparisons (Al Adhami *et al* and Duteil *et al* datasets) are shown on the *x* and *y*-axis, respectively.

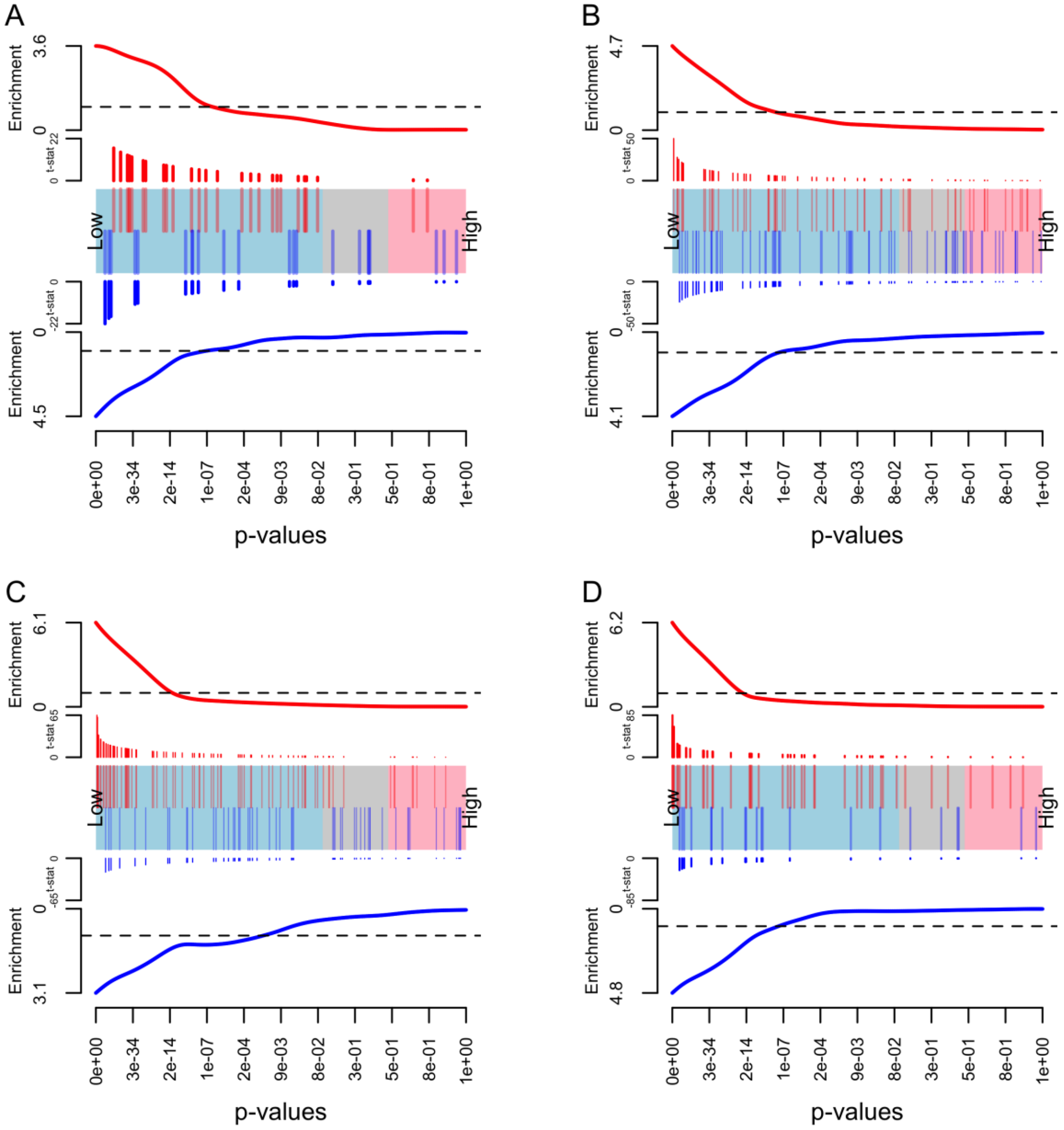

Supplementary Figure 2: Enrichment of signaling pathways at differentiated adipocytes. Bar-codes plots of significant enrichment of signaling pathways; mTOR (**A**) Jak-STAT (**B**) Insulin (**C**) and adipocytokine signaling pathway (**D**). *p*-values are shown on the x-axis ordered from high to low and divided by the cutoffs 0.01 and 0.05, respectively. *p*-values and *t*-statistics are calculated for the comparison between differentiating (day 6 after treatment) and proliferating cells (day 0). The vertical bars represent the individual genes with their corresponding *t*-statistics; *red* line (positive) and *blue* line (negative). The horizontal lines are the overall enrichment of the pathway.
